# Intraneural Injection of ATP Stimulates Regeneration of Primary Sensory Axons in the Spinal Cord

**DOI:** 10.1523/JNEUROSCI.1660-17.2017

**Published:** 2018-02-07

**Authors:** Dongsheng Wu, Sena Lee, Juan Luo, Haijian Xia, Svetlana Gushchina, Peter M. Richardson, John Yeh, Ute Krügel, Heike Franke, Yi Zhang, Xuenong Bo

**Affiliations:** ^1^Centre for Neuroscience and Trauma, Blizard Institute, Barts and the London School of Medicine and Dentistry, Queen Mary University of London, London E1 2AT, United Kingdom,; ^2^Department of Neurosurgery, Royal London Hospital, London E1 1BB, United Kingdom, and; ^3^Rudolf Boehm Institute of Pharmacology and Toxicology, Medical Faculty, Universität Leipzig, D-04107, Leipzig, Germany

**Keywords:** ATP, axon regeneration, conditioning injury, purinergic, sensory neurons, spinal cord injury

## Abstract

Injury to the peripheral axons of sensory neurons strongly enhances the regeneration of their central axons in the spinal cord. It remains unclear on what molecules that initiate such conditioning effect. Because ATP is released extracellularly by nerve and other tissue injury, we hypothesize that injection of ATP into a peripheral nerve might mimic the stimulatory effect of nerve injury on the regenerative state of the primary sensory neurons. We found that a single injection of 6 μl of 150 μm ATP into female rat sciatic nerve quadrupled the number of axons growing into a lesion epicenter in spinal cord after a concomitant dorsal column transection. A second boost ATP injection 1 week after the first one markedly reinforced the stimulatory effect of a single injection. Single ATP injection increased expression of phospho-STAT3 and GAP43, two markers of regenerative activity, in sensory neurons. Double ATP injections sustained the activation of phospho-STAT3 and GAP43, which may account for the marked axonal growth across the lesion epicenter. Similar studies performed on P2X7 or P2Y2 receptor knock-out mice indicate P2Y2 receptors are involved in the activation of STAT3 after ATP injection or conditioning lesion, whereas P2X7 receptors are not. Injection of ATP at 150 μm caused little Wallerian degeneration and behavioral tests showed no significant long-term adverse effects on sciatic nerve functions. The results in this study reveal possible mechanisms underlying the stimulation of regenerative programs and suggest a practical strategy for stimulating axonal regeneration following spinal cord injury.

**SIGNIFICANCE STATEMENT** Injury of peripheral axons of sensory neurons has been known to strongly enhance the regeneration of their central axons in the spinal cord. In this study, we found that injection of ATP into a peripheral nerve can mimic the effect of peripheral nerve injury and significantly increase the number of sensory axons growing across lesion epicenter in the spinal cord. ATP injection increased expression of several markers for regenerative activity in sensory neurons, including phospho-STAT3 and GAP43. ATP injection did not cause significant long-term adverse effects on the functions of the injected nerve. These results may lead to clinically applicable strategies for enhancing neuronal responses that support regeneration of injured axons.

## Introduction

Weak intrinsic regenerative capacity of injured neurons in the adult mammalian CNS is a major obstacle for neural repair. Efficacious approaches to elevate the regenerative state of injured neurons are considered to be crucial in achieving successful axonal regeneration (He and Jin, 2016). It has been observed that an injury to the peripheral axons of the primary sensory neurons can increase their intrinsic growth capacity (McQuarrie and Grafstein, 1973) and strongly enhances the regeneration of their central branches in the spinal cord (Richardson and Issa, 1984; Richardson and Verge, 1987; Neumann and Woolf, 1999), a phenomenon termed conditioning lesion. Although the exact mechanism of conditioning lesion is still under debate, strong evidence indicates that neuropoietic cytokines, especially, ciliary neurotrophic factor (CNTF), leukemia inhibitory factor (LIF), and interleukin-6 (IL-6), play an important role (Snider et al., 2002; Raivich and Makwana, 2007; Richardson et al., 2009). Released cytokines induce phosphorylation of signal transducer and activator of transcription 3 (STAT3; Lee et al., 2004; Qiu et al., 2005; Wu et al., 2009), which switches on the transcription of growth related molecules in injured neurons. cJun and activating transcription factor 3 (ATF3) are activated in dorsal root ganglion (DRG) neurons as well (Herdegen et al., 1992; Tsujino et al., 2000). cAMP signaling pathway is also implicated in the mechanism of conditioning lesion (Qiu et al., 2002). It is evident that the increased growth status of neurons after peripheral nerve injury is mediated coordinately by multiple signaling pathways.

How nerve injury triggers the molecular events that elevate the regenerative state of sensory neurons remains unclear (Richardson et al., 2009). Better understanding of the molecular events underlying this phenomenon could lead to practical methods to boost the intrinsic regenerative capacity. Extracellular ATP has a broad range of biological activities including glia-glia and glia-neuron communication (Burnstock, 2007; Abbracchio et al., 2009). ATP is released extracellularly by nerve or other tissue injury (Fields and Burnstock, 2006) and acts as an injury signal in various tissues (Rodrigues et al., 2015). We postulate that ATP released by nerve injury triggers a series of molecular events in Schwann cells and/or axons, leading to the activation of regenerative machinery in the injured neurons. If that were the case, injection of ATP into a peripheral nerve should be able to mimic the effect of nerve injury in promoting the regeneration of the central branches of sensory neurons. In this study, we tested our theory using both *in vitro* and *in vivo* axonal growth models and examined the activation of several growth-related transcription factors and molecules in DRG neurons after ATP injection.

## Materials and Methods

### 

#### 

##### Animals and surgical procedures.

Animal experiments were conducted in accordance with the UK Animals (Scientific Procedures) Act 1986 under project license from the Home Office or approved by the regional governmental agency of the State of Saxony, Germany. Female Wister rats (200–250 g, 6–8 rats per group) were anesthetized with isoflurane. The spinal cord was exposed via dorsal laminectomy of the eighth thoracic vertebra and the dura was opened. Subsequently, bilateral dorsal column funiculus was transected with a pair of microscissors to a depth of 1.5 mm from the dorsal surface, and a 29 gauge needle was used to plow the lesion site to assure the complete transection of the dorsal column. The dura, muscle, and skin were then sutured. Immediately after dorsal column transection the left sciatic nerve at mid-thigh level was exposed and 6 μl of 150 μm ATP was injected into the nerve using a 31 gauge Hamilton steel needle. The needle was left in place for 1 min and then gradually withdrawn to prevent leakage from the needle track. Three control groups were used: (1) injection of 6 μl of 0.9% NaCl (saline) into sciatic nerve, (2) crush of left sciatic nerve with a jeweler's forceps for 15 s at mid-thigh level, and (3) sham-operation with the exposure of sciatic nerve only. The experimental procedures for double sciatic nerve treatments are described in the Results. Animals were allowed to survive for 6 weeks after surgery. Three days before perfusion of the animals, 5 μl of 1% cholera toxin B subunit (CTB; List Biological Laboratories) was injected into the left sciatic nerve at hip level to trace the axons.

To identify the purinoceptor subtypes involved in promoting axonal growth after conditioning lesion and ATP injection, we also performed sciatic nerve injection and crush in P2X7 receptor knock-out (P2X7R_KO) and P2Y2 receptor knock-out (P2Y2R_KO) mice. Homozygous P2X7R_KO (P2X7R^−/−^) mice line was a generous gift from GSK plc, which was bred to a C57BL/6 background (Chessell et al., 2005). We confirmed the P2X7R deficiency by genotyping and immunostaining of thymus, lungs, and sciatic nerves with anti-P2X7R antibody (Luo et al., 2013). P2Y2R_KO mice were originally generated on a B6D2 background (a hybrid between female C57BL/6 and male DBA/2 mice) and further crossbred with 129Sv mice in Beverley Koller's laboratory (University of North Carolina; Homolya et al., 1999). At the Rudolf Boehm Institute of Pharmacology and Toxicology, Leipzig, the P2Y2R_KO colony was backcrossed to 129S2/SvHsd for a total of nine generations and mating of heterozygous animals provided P2Y2R_KO and wild-type littermates. We confirmed the P2Y2R deficiency by genotyping. The surgical procedures were performed in this institute. The sciatic nerve crush and injection procedures on mice were similar to those for rats. Female mice (20–25 g) were anesthetized with isoflurane, 2 μl of saline, ATP, or UTP was injected into the left sciatic nerve at mid-thigh level using a 31 gauge Hamilton steel needle.

##### Neurite outgrowth assay.

Four groups of rats (*n* = 4 per group) were subjected to sham operation, sciatic nerve crush, injection of 6 μl of 150 μm ATP or 6 μl of saline. Three days after the surgery, rats were killed and ipsilateral L4 and L5 DRG were removed and digested with 0.125% collagenase (type XI, Sigma-Aldrich) and triturated in 1 ml BSF2 medium [F-12/DMEM, N2 supplement (Thermo Fisher), 0.3% bovine serum albumin (BSA)], and penicillin/streptomycin) and centrifuged through a cushion of 2 ml 15% BSA at 100 × *g* for 5 min. The cell pellet was resuspended in BSF2 and the dissociated neurons were plated at 1000 cells per well in 8-well chamber slides precoated with poly-l-lysine and laminin (0.1 μg per well, Thermo Fisher). Neurons were fixed 16 h after seeding and immunostained with a mouse monoclonal β tubulin III antibody (see Table 1 for antibody information). The length of the longest neurite of each neuron was measured using ImageJ (https://imagej.nih.gov/ij/). The percentages of neurons with neurites longer than the diameter of their somata were also counted. Two wells of DRG neuron cultures per animal and 200–400 neurons per well were quantified.

**Table 1.** Antibodies used

Antibody	Dilution factor	Company	Catalog No.	RRID
Phospho-STAT3 (Tyr705)	1:200	Cell Signaling Technology	9131	AB_331586
Phospho-c-Jun (Ser63) (54B3)	1:200	Cell Signaling Technology	2361	AB_490908
ATF3	1:500	Santa Cruz Biotechnology	Sc-188	AB_2258513
GAP43	1:10,000	Atlas Antibodies	HPA015600	AB_1234436
β-Tubulin III	1:400	Sigma-Aldrich	T8660	AB_477590
Myelin protein P0	1:3000	ASTEXX		
NF 200	1:2000	Sigma-Aldrich	N4142	AB_477272
p75^NTR^	1:2000	Millipore	ABN1655	
S100	1:2000	Sigma-Aldrich	S2657	AB_261477
GFAP	1:1000	Millipore	MAB360	AB_2109815
CTB	1:4000	List Biological	703	AB_10013220
Goat anti-rabbit IgG_AlexaFluor 568	1:400	Thermo Fisher Scientific	A11036	AB_10563566
Donkey anti-sheep IgG_AlexaFluor 568	1:1000	Thermo Fisher Scientific	A21099	AB_10055702
Goat anti-rabbit IgG_AlexaFluor 488	1:400	Thermo Fisher Scientific	A11034	AB_2576217
Goat anti-mouse IgG_AlexaFluor 568	1:400	Thermo Fisher Scientific	A11031	AB_144696
Biotinylated donkey anti-goat IgG	1:400	Jackson ImmunoResearch	705-065-003	AB_2340396
FITC-conjugated donkey anti-mouse IgG	1:400	Jackson ImmunoResearch	715-096-150	AB_2340795

##### Immunohistochemistry.

Detailed information on the antibodies used in this study is listed in Table 1. For quantification of regenerating axons after dorsal column transection, animals were perfused 6 weeks after injury. A 10 mm lesion block centered at the lesion site was removed and sagittal longitudinal sections of 12 μm thick were cut and mounted on a series of slides (see Fig. 2*A*, describes the orientation of the sections). For identification of CTB-labeled axons in dorsal column and lesion epicenter, sections were immunostained with goat anti-CTB antibody and mouse anti-glial fibrillary acidic protein (GFAP), followed by incubation with biotinylated donkey anti-goat IgG and FITC-conjugated donkey anti-mouse IgG. Sections were then incubated in streptavidin AlexaFluor 568. Photomicrographs were taken using a digital camera attached to a Leica DM5000 epifluorescence microscope. Confocal microscopy images of CTB-labeled axons in the spinal cord lesion epicenter were captured by a Zeiss LSM 710 confocal microscope using a 63× oil objective. Images were taken at a *z*-stack of 34 sections with section interval of 0.348 μm. Each series of *z*-stack images was processed with a maximum intensity projection tool to create a final image with pixels containing the maximum value at the whole stack.

CTB-labeled axons in the white matters were counted in six to seven sagittal sections at 36 μm intervals encompassing all CTB-labeled dorsal column axons. For the experiment with single ATP or saline injections, as no axons grew across the lesion epicenter, we counted the numbers of CTB-labeled axons at three distance points: 0.35, 1.05, and 1.75 mm caudal to the caudal border of the lesion epicenter, and those axons growing into the lesion epicenter in a sagittal longitudinal section. For the double-injection experiment, the lesion epicenter was divided into three equal regions: caudal, central, and rostral. CTB-labeled axons growing across the lesion epicenter into the rostral region with reactive astrocyte processes immediately adjacent to the lesion epicenter were counted in 3 distance points: 0.35, 1.05, and 1.75 mm rostral to the arbitrary boundary defined by GFAP immunoreactivity.

For detecting the expression of the activated transcription factors (pSTAT3, pcJun, and ATF3) and GAP43 in rat and mouse L4–L5 DRG, DRG sections were incubated with rabbit anti-phospho-STAT3 (Tyr705), rabbit anti-phospho-cJun, rabbit anti-ATF3, or rabbit anti-GAP43, followed by incubation with goat anti-rabbit IgG AlexaFluor 568 conjugate. The nuclei were stained with 4,6-diamidino-2-phenylindole (DAPI). The proportion of DRG neurons with activated transcription factors or GAP43 was determined by analyzing 300–500 neurons with visible nuclear profiles (by DAPI staining) per DRG in randomly chosen sections.

To examine the histological change of sciatic nerves after nerve crushes or injections of ATP, animals were perfused 3 d after the treatments. Longitudinal sections (8 μm thick) were double-stained with mouse anti-myelin protein P0 antibody plus rabbit anti-neurofilament 200 (NF200) antibody, or mouse anti-S100 antibody plus rabbit anti-p75^NTR^ antibody overnight at room temperature, followed by incubation with AlexaFluor 568-conjugated goat anti-mouse IgG antibody and goat anti-rabbit IgG AlexaFluor 488-conjugated antibody for 2 h. Images were taken at three regions from the sciatic nerve: at the center of the crush or injection site (the central region), 1.4 mm proximal and 1.4 mm distal to the crush or injection site. From each animal, four images from each region were taken for quantification. Integrated densities of P0, NF200, S100, and p75^NTR^ immunoreactivity were measured with ImageJ.

##### ELISA.

To investigate whether ATP injection can induce the expression of IL-6, we measured the levels of IL-6 in DRG and sciatic nerves. Three days after single sciatic nerve treatments (3 rats per group), left L4–L5 DRG and a 1 cm segment of left sciatic nerve encompassing the injection or crush site were removed. Segments of sciatic nerves corresponding to the same positions as those of treated sciatic nerves were also removed from the sham-operated animals. Soluble proteins were extracted from the tissues and protein concentrations were measured using a DC Protein Assay kit (Bio-Rad). Concentrations of IL-6 were measured using a Quantikine ELISA kit (R&D Systems) according to the instruction of the kit.

##### Tests for sciatic nerve function.

To investigate whether double intraneural injection of ATP has any adverse effect on sciatic never functions we performed behavioral tests on sensory and motor functions. Female Wistar rats (200 g) were divided into four groups (5 per group): sham-operated control, intraneural injection of saline/saline, ATP/saline, and ATP/ATP. The animals were accommodated to the behavioral equipment for 10 min every day for 1 week before the tests. Tests for baseline were performed 3 d before the first intraneural injection. Behavioral tests were performed on days 2 and 6 after the first injection. The second intraneural injection was performed on day 7, and behavioral tests were continued on day 9, 13, 16, and 20. The experimenter was blind to treatment groups throughout the testing period.

Mechanical sensitivity was assessed by measuring paw withdrawal threshold using Von Frey hairs according to the “up-down” method as previously described (Dixon, 1980; Chaplan et al., 1994). Noxious thermal sensitivity was assessed using the Hargreaves' method (Hargreaves et al., 1988), by measuring the time taken for a radiant heat source to elicit a flexion reflex. For footprint analysis, rat hindpaws were covered with ink to record walking patterns during continuous locomotion across a wooden runway (8 × 120 cm). The hindpaw first–fifth toe spread was measured and used as an indicator of functional recovery of injured sciatic nerve (Walker et al., 1994). In the grid walking test rats were trained to cross a wire grid (40 × 65 cm with 5 × 5 cm grid squares). Hindlimb slips were recorded (determined by a paw slipping below the plane of the grid) among the first 30 steps.

##### Experimental design and statistical analysis.

The details for quantification of neurites, transcription factors and GAP43, and CTB-labeled axons in the spinal cord are described in the relevant sections above. Statistical analyses for neurite outgrowth, rat DRG neurons expressing activated transcription factors or GAP43, IL-6 levels, CTB-labeled axons among different groups, and the quantification data of sciatic nerve markers were conducted using one-way ANOVA with *post hoc* Tukey tests in SPSS software. Two-way ANOVA with *post hoc* Tukey tests were used for analyzing the behavioral data to compare the changes before and after the treatments of the same groups and among different groups, for analyzing the DRG neurons expressing activated transcription factors or GAP43 in P2X7R_KO or P2Y2R_KO mice and the wild-type mice of the same genetic background. All data are shown as mean ± SEM.

## Results

### Single intraneural ATP injection enhances neurite outgrowth of DRG neurons

**Figure 1.**
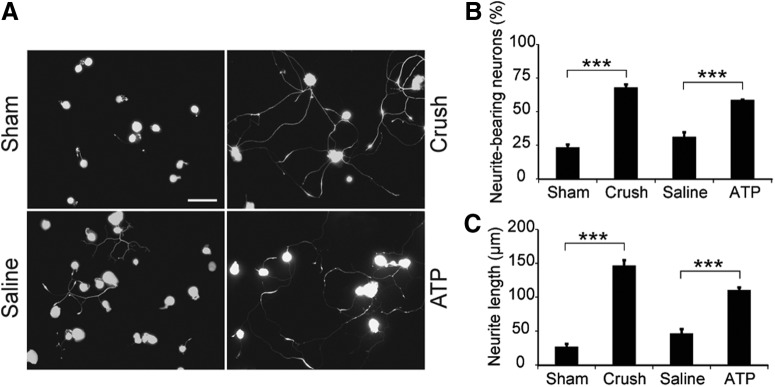
Intraneural injection of ATP enhances neurite outgrowth of DRG neurons. ***A***, Cultured DRG neurons immunostained with an anti-βIII tubulin antibody showing the neurite growth from four experimental groups: sham operation, sciatic nerve crush, sciatic nerve injection of saline or ATP. Scale bar, 100 μm. ***B***, ***C***, Quantification of the percentages of neurite-bearing neurons (***B***) and neurite lengths (***C***) in the four groups. ***p < 0.001. *n* = 4, one-way ANOVA with *post hoc* Tukey test.

To prove our hypothesis that sciatic nerve injection of ATP can mimic the effect of a conditioning lesion, we first investigated whether injection of ATP into rat sciatic nerves could promote the neurite outgrowth of DRG neurons dissociated and maintained *in vitro*. Four groups of adult rats were subjected to sham-operation, sciatic nerve crush, normal saline injection, or ATP (150 μm) injection and the DRG neurons were isolated from the animals 3 d later. After 16 h in culture, DRG neurons grew neurites to different lengths (Fig. 1*A*) and 23 ± 2% of DRG neurons in the sham-operated group grew neurites longer than the diameters of their cell bodies (Fig. 1*B*). Sciatic nerve crush significantly increased the percentage of neurite-bearing cells to 68 ± 2% (*p* = 2.7 × 10^−8^ compared with sham-operated group, *n* = 5). In the saline-injected group the proportion of the neurite-bearing neurons was 31 ± 3%, which is not significantly different from the sham-operated group. Injection of ATP increased the neurite-bearing cells to 57 ± 2%, which is significantly higher than the saline-injected group (*p* = 5.1 × 10^−5^, *n* = 5). A similar pattern of results was obtained by measuring the longest neurite of each neuron (Fig. 1*C*). Neurons from the sciatic nerve crush group had much longer neurites than those from sham-operated group (147 ± 8 vs 27 ± 4 μm; *p* = 2.1 × 10^−9^, *n* = 5). In the saline-injected group, the neurite length was 47 ± 7 μm, which was not significantly longer than that of the sham operated group, but significantly shorter than the ATP-injected group (111 ± 4 μm; *p* = 1.5 × 10^−5^, *n* = 5). The results show that a single intraneural ATP injection elevates the regeneration capacity of DRG neurons and promotes neurite outgrowth.

### Single intraneural ATP injection enhances sensory axonal regeneration in spinal cord

**Figure 2.**
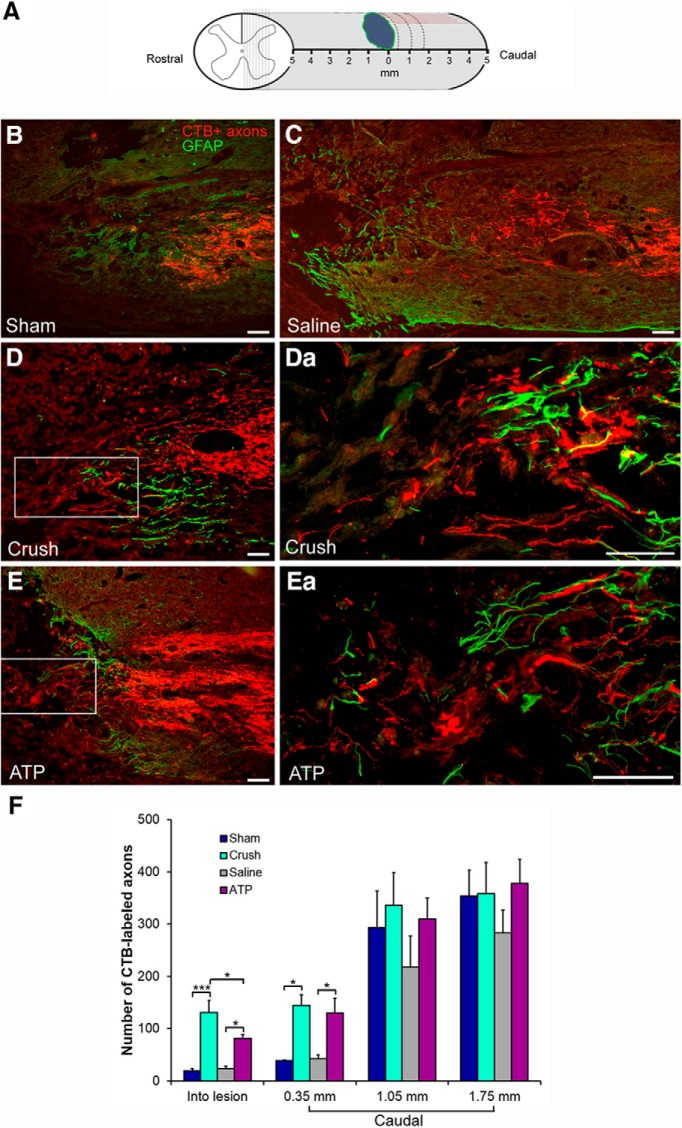
Intraneural injection of ATP promotes axonal regeneration after dorsal column transection. ***A***, Scheme showing the spinal cord segment containing the T8 dorsal column lesion. Light gray lines indicate the sagittal sections through the dorsal column. Green irregular line indicates the glial scar surrounding the lesion epicenter. Red lines represent CTB-labeled axons. Dotted lines indicate the three distance points for counting the CTB-labeled axons. ***B***–***E***, Photomicrographs showing CTB-labeled axons (red) growing toward the caudal lesion border (defined by GFAP immunoreactivity, green) or into the lesion epicenter in spinal cord sagittal sections from the 4 groups. The boxed areas in ***D*** and ***E*** are shown at higher-magnification in ***Da*** and ***Ea***. Scale bars, 100 μm. ***F***, Quantification of CTB-labeled axon numbers in the lesion epicenter and caudal to the lesion epicenter. *p < 0.05, ***p < 0.001. *n* = 6, one-way ANOVA with *post hoc* Tukey test.

To investigate whether the axon-growth promoting effect of intraneural ATP injection can be achieved *in vivo*, the dorsal column of the spinal cord was transected bilaterally at T8 in adult rats, which were subjected to one of the four treatments to the sciatic nerves described above. Transganglionic tracer CTB was used to label the ascending sensory axons. Six weeks after surgery, animals were killed and spinal cord segments containing the lesion site were sectioned and stained with an anti-CTB antibody. CTB-labeled axons were clearly visible in sagittal spinal sections containing the lesion sites in all four groups (Fig. 2*B*). End-bulb structures could be seen at various distances caudal to the lesion epicenter, which were most abundant in the area within 2 mm of the caudal border of the lesion epicenter. For statistical analysis three points caudal to the border of lesion epicenter (defined by GFAP immunoreactivity) were arbitrarily selected and the CTB-labeled axons at each distance point and those inside the lesion epicenter were counted (Fig. 2*A*,*F*). At 1.75 and 1.05 mm caudal to the border of the lesion epicenter, the numbers of CTB-labeled axons did not differ significantly among the four groups (Fig. 2*F*). However, more CTB-labeled axons were found at 0.35 mm caudal to the epicenter border or within the lesion epicenter in the nerve crush and the ATP-injected groups than the sham-operated and saline-injected groups (Fig. 2*C*). At 0.35 mm to the caudal border of lesion epicenter, 35 ± 3 CTB-labeled axons per animal were counted in the sham-operated group, whereas 144 ± 21 were seen in the sciatic nerve crush group. In the saline injection group, 42 ± 8 CTB-labeled axons were counted, which is similar to the sham-operated group, but significantly lower than the ATP injection group (130 ± 25; *p* = 0.027, *n* = 6; Fig. 2*C*). No significant difference in CTB-labeled axon numbers was seen between the sciatic nerve crush and the ATP injection group. Inside the lesion epicenter few CTB-labeled axons were found in the sham-operated (19 ± 4) and saline injection groups (23 ± 5), whereas significantly more CTB-labeled axons were counted in the lesion epicenter in the sciatic nerve crush group (131 ± 23; *p* = 1.5 × 10^−5^ compared with the sham-operated group, *n* = 6). Also the number of CTB-labeled axons in the lesion epicenter in the ATP injection group (96 ± 16) was significantly higher than the saline injection group (*p* = 0.0014, *n* = 6), but lower than the nerve crush group (*p* = 0.049, *n* = 6). The data from this *in vivo* study demonstrate that ATP injection into a peripheral nerve is able to promote the regeneration of the corresponding central sensory axons.

### Double intraneural ATP injection markedly enforces the stimulatory effect of a single ATP injection

**Figure 3.**
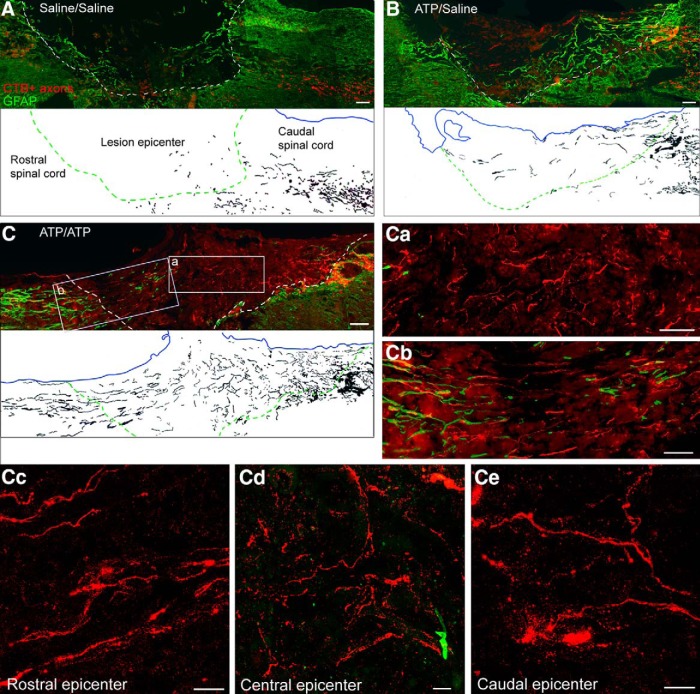
Double intraneural injection of ATP promotes profound axonal regeneration after dorsal column transection. ***A***–***C***, Montages of photomicrographs showing CTB-labeled sensory axons (red) growing into or across the lesion epicenter (defined by green GFAP immunoreactivity, and marked by dashed lines) in sagittal sections of spinal cord from saline/saline (***A***), ATP/saline (***B***), and ATP/ATP (***C***) double-injection groups. Camera lucida drawings corresponding to the same micrographs are used to map the CTB-labeled axons inside and around the lesion epicenters. Scale bar, 100 μm. Boxed areas in ***C*** are enlarged in ***Ca*** and ***Cb***. Scale bar, 50 μm. ***Cc***–***Ce***, *Z*-stack confocal microscopy images showing CTB-labeled axons in the rostral (100 μm from the rostral boundary line), central, and caudal (200 μm from the caudal boundary line) regions of the lesion epicenter in a section 36 μm from the section shown in ***C***. Scale bar, 10 μm.

**Figure 4.**
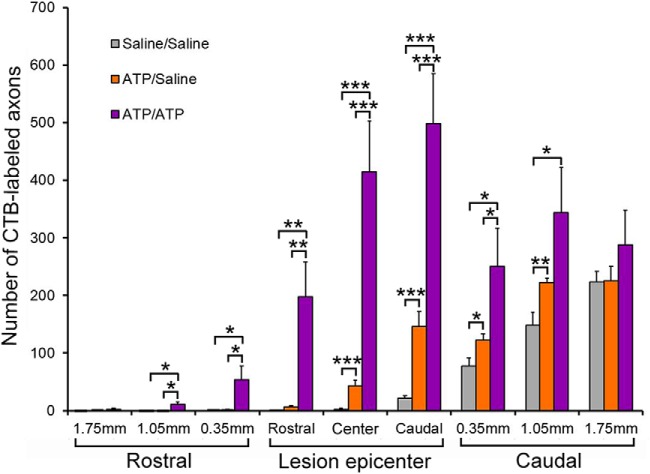
Quantification of the numbers of CTB-labeled axons. CTB-labeled axons were counted in three equally divided regions within the lesion epicenter and in 3 regions on both the rostral and caudal sides immediately adjacent to the lesion epicenter in six to seven sagittal sections at 36 μm intervals. The distances in both the caudal and rostral spinal cord were measured from the arbitrary boundaries defined by GFAP-immunoreactive astrocyte processes. *p < 0.05, **p < 0.01, ***p < 0.001. *n* = 8, one-way ANOVA with *post hoc* Tukey test.

In view of a report that a second peripheral nerve transection significantly increases the enhancement of central sensory axonal regeneration compared with a single nerve transection (Neumann et al., 2005), we assessed the effects of a second ATP injection 1 week after the first one. Three groups of animals were subjected to dorsal column transection: one group underwent two intraneural ATP injections 1 week apart (ATP/ATP group); the second group underwent ATP injection, followed by saline injection 1 week later (ATP/saline group); and the third group underwent two saline injections (saline/saline group). All the other experimental procedures were the same as those of single treatment experiments. CTB-labeled axons were counted in the spinal cord at various distances caudal or rostral to the lesion epicenter as well as at three regions inside the lesion epicenter: caudal, central, and rostral (see Fig. 4). As it is shown in the montaged fluorescence images and their corresponding camera lucida drawings (Fig. 3*A–C*), in the saline/saline group a small number of CTB-labeled axons grew into the caudal region of lesion epicenter (22 ± 4; Fig. 4), and only a few axons grew into the central region of lesion epicenter, but no axon grew to the rostral region of the lesion epicenter (Fig. 3*A*). In the ATP/saline group, more axons entered the lesion epicenter than in the saline/saline group (Fig. 4), and a few CTB-labeled axons extended to the rostral region of lesion epicenter (Fig. 3*B*). The counts of CTB-labeled axons were 146 ± 25, 43 ± 10, and 7 ± 3 in the caudal, central, and the rostral regions inside the lesion, respectively. In the ATP/ATP group, a large number of CTB-labeled axons grew across the lesion epicenter and some of them entered the rostral spinal cord containing hypertrophic astrocyte processes immediately adjacent to the lesion epicenter (Fig. 3*C*). In the central region of the lesion epicenter, the CTB-labeled regenerating axons looked disorderly and had many branches (Fig. 3*Ca*), however, when they approached the rostral border they were more aligned (Fig. 3*Cb*). The morphology of the CTB-labeled axons inside lesion epicenter is more clearly shown in the *z*-stack confocal microscopy images (Fig. 3*Cc–Ce*). The CTB-labeled axon counts in the caudal, central, and rostral regions in the lesion epicenter were 498 ± 88, 415 ± 88, and 198 ± 60, respectively (Fig. 4). The number of axons growing into the lesion epicenter in the ATP/ATP group was ∼10-fold more than that in ATP/saline group, and 166-fold more than that in the saline/saline group. A few regenerating axons extended as far as 1.75 mm into the rostral spinal cord from the arbitrary border defined by reactive astrocytes in the ATP/ATP group, whereas no axon in the other two groups entered the area. These data show that a second ATP injection markedly reinforces the stimulatory effect of the first ATP injection on axonal regeneration.

### Single intraneural ATP injection increases the expression of IL-6 in DRG and sciatic nerves

**Figure 5.**
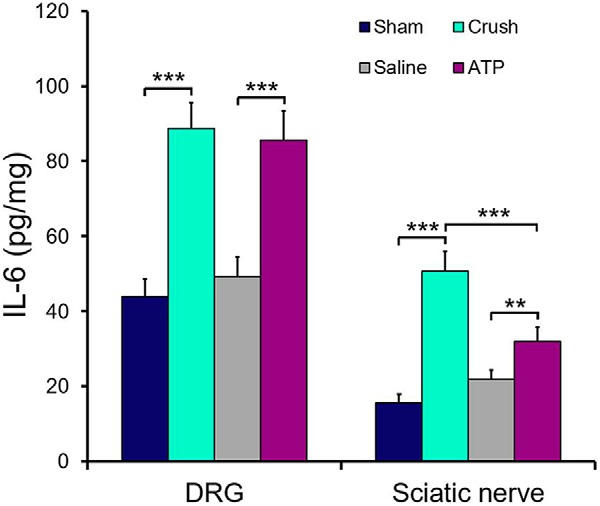
Single intraneural injection of ATP increases the concentrations of IL-6 in DRG and sciatic nerves. Sandwiched ELISA was used to measure the levels of IL-6 in protein extracts from the treated segments of sciatic nerves and DRG from the rats subjected to sciatic nerve crush, injections of saline or 150 μm ATP. Nerves were taken 3 d after treatments. The segments of sciatic nerves corresponding to those segments of the treated nerves were taken from the sham operated rats and used as control. **p < 0.01, ***p < 0.001. *n* = 3, one-way ANOVA with *post hoc* Tukey test.

IL-6 is considered as a major mediator in the growth promoting effects of conditioning lesion. Our ELISA results showed that both sciatic nerve and DRG had base level expression of IL-6 and the level of IL-6 in DRG was higher than sciatic nerves (Fig. 5). Nerve crush or a single injection of 6 μl 150 μm ATP significantly increased the IL-6 concentrations in DRG and sciatic nerves 3 d after treatments, whereas saline injection did not (Fig. 5). Thus, the results show that a single ATP injection into sciatic nerve has a similar effect to nerve crush in inducing IL-6 expression.

### Single intraneural ATP injection induces the activation of transcription factors and expression of GAP43

**Figure 6.**
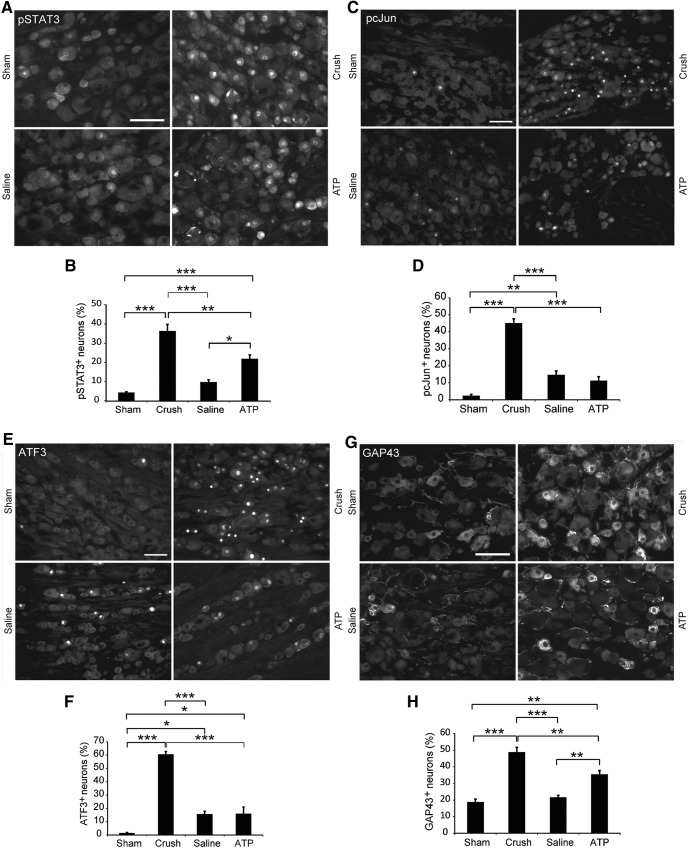
Single intraneural ATP injection induces activation of transcription factors and expression of GAP43 in DRG neurons. Four groups of rats were subjected to sham operation, sciatic nerve crush, injections of saline or ATP into sciatic nerves. DRG were removed 3 d after the treatments and processed for immunohistochemistry. ***A***, ***C***, ***E***, ***G***, Micrographs showing the immunoreactivity for pSTAT3 (***A***), pcJun (***C***), ATF3 (***E***), and GAP43 (***G***) in DRG neurons. Scale bar, 100 μm. ***B***, ***D***, ***F***, ***H***, Quantification of neurons with nuclear profiles containing immunoreactivity for pSTAT3 (***B***), pcJun (***D***), ATF3 (***F***), and GAP43 (***H***). *p < 0.05, **p < 0.01, ***p < 0.001. *n* = 4, one-way ANOVA with *post hoc* Tukey test.

Because a conditioning lesion induces the expression or activation of several transcription factors associated with the regenerative status of the primary sensory neurons, we also explored ATP-induced changes of these molecular markers. We first examined whether ATP injection into a peripheral nerve could activate the STAT3 signaling pathway. Three days after the four single treatments described above, L4–L5 DRG were removed and immunostained for pSTAT3. In DRG from sham-operated animals, only 4 ± 1% neurons were positive for pSTAT3, whereas sciatic nerve crush increased pSTAT3^+^ neurons to 36 ± 4% (Fig. 6*A*,*B*). Saline injection also caused moderate increase in pSTAT3^+^ neurons (10 ± 1%), but not significantly higher than the sham-operated group. Injection of ATP led to an increase of pSTAT3^+^ neurons to 21 ± 2%, which was significantly higher than the saline injection group (*p* = 0.01, *n* = 4), but significantly lower than the sciatic nerve crush group (*p* = 0.003, *n* = 4). The results demonstrate that intraneural injection of ATP can activate the STAT3 signaling pathway, although not as potent as nerve crush.

The other two transcription factors examined were pcJun (Fig. 6*C*,*D*) and ATF3 (Fig. 6*E*,*F*). In sham-operated animals, the numbers of both ATF3^+^ and pcJun^+^ neurons in DRG were rather low (1.5 ± 0.4% and 2 ± 1%, respectively), indicating that the procedure of exposing the nerve did not induce injury responses in the neuronal cell bodies. Nerve crush increased the ATF3^+^ neurons to 60 ± 2% and pcJun to 45 ± 3%. Single saline or ATP injection moderately increased the percentages of ATF3^+^ (15 ± 2% and 15 ± 5%, respectively) and pcJun^+^ (15 ± 2% and 11 ± 2%, respectively) neurons; however, there was no significant difference between saline and ATP injection groups, indicating that the increased expression of ATF3 and pcJun was caused by the injection procedure.

GAP43 is also considered as a marker for neurons' propensity for axonal regeneration (Schreyer and Skene, 1991; Hu-Tsai et al., 1994; Liu et al., 2011). Various levels of GAP43 immunoreactivity were observed in the cytoplasm of 19 ± 2% DRG neurons in sham-operated animals (Fig. 6*G*,*H*). Sciatic nerve crush significantly increased the number of GAP43^+^ DRG neurons (49 ± 3%). In the saline injection group 22 ± 1% neurons were GAP43^+^, which was not significantly higher than the sham-operated animals. ATP injection increased the percentage of GAP43^+^ neurons to 35 ± 2%, which is significantly higher than the saline-injected animals (*p* = 0.004, *n* = 4), but lower than the nerve crush group (*p* = 0.005, *n* = 4). Thus, single ATP sciatic nerve injection can increase the expression of GAP43 in DRG neurons, although to a lower extent compared with nerve crush.

### Double intraneural ATP injection sustains the activation of transcription factors and the expression of GAP43

**Figure 7.**
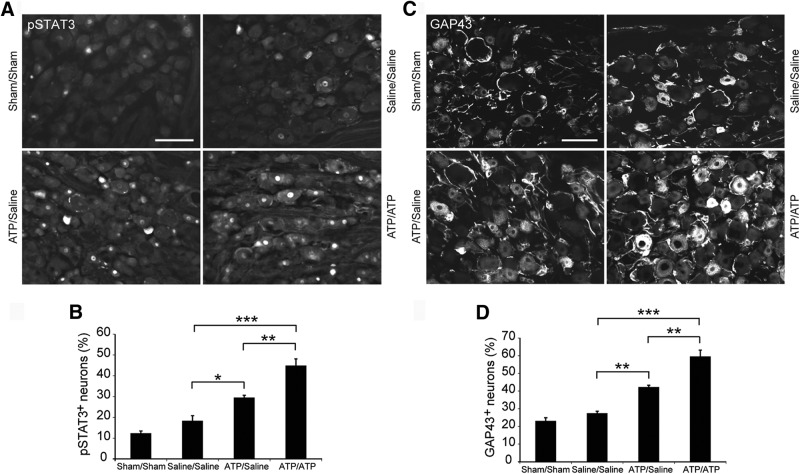
Double intraneural ATP injection sustains activation of STAT3 and expression of GAP43 in DRG neurons. Four groups of rats were subjected to sham/sham operation, saline/saline injection, ATP/saline injection, and ATP/ATP injection into sciatic nerves. DRG were removed 20 d after the first treatments for immunohistochemistry. ***A***, ***C***, Photomicrographs showing the immunoreactivity for pSTAT3 (***A***) and GAP43 (***C***) in DRG neurons. Scale bar, 100 μm. ***B***, ***D***, Quantification of neurons with nuclear profiles containing immunoreactivity for pSTAT3 (***B***) and GAP43 (***D***). *p < 0.05, **p < 0.01, ***p < 0.001. *n* = 3, one-way ANOVA with *post hoc* Tukey test.

To investigate whether a second ATP injection can boost and sustain the expression of pSTAT3 and GAP43 in DRG neurons, four groups of rats were subjected to double sham operations (sham/sham), saline/saline, ATP/saline, and ATP/ATP double injections 1 week apart. Animals were maintained for 20 d after the initial injection before they were killed. Percentages of pSTAT3^+^ neurons in DRG were 12 ± 1%, 20 ± 3%, 30 ± 1%, and 45 ± 3% in sham/sham, saline/saline, ATP/saline, and ATP/ATP groups, respectively (Fig. 7*A*,*B*). There was no significant difference between sham/sham and saline/saline groups. The ATP/saline group has significantly more pSTAT3^+^ neurons than the saline/saline group (*p* = 0.04, *n* = 3), whereas the ATP/ATP group has more pSTAT3^+^ neurons than both saline/saline and ATP/saline groups (*p* = 0.0001 and 0.006, respectively, *n* = 3). The results suggest that sustained activation of STAT3 by double ATP injection may contribute to the significantly enhanced axonal growth in the lesioned spinal cord shown above.

We also examined the GAP43 expression in DRG from the four groups of rats with double treatments. The percentages of GAP43^+^ DRG neurons were 23 ± 2%, 27 ± 1%, 42 ± 1%, and 60 ± 4% in sham-operated, saline/saline-injected, ATP/saline-injected, and ATP/ATP-injected groups, respectively (Fig. 7*C*,*D*). The ATP/saline group had significantly more GAP43^+^ neurons than saline/saline group (*p* = 0.008, *n* = 3); however, the percentage of GAP43^+^ neurons in ATP/ATP group was significantly higher than both saline/saline (*p* = 1.3 × 10^−5^, *n* = 3) and ATP/saline group (*p* = 0.003, *n* = 3), suggesting that more DRG neurons entered and maintained in regenerative state after the second ATP injection.

### Knocking out P2X7 receptor has no effects on the expression of axon growth associated proteins

**Figure 8.**
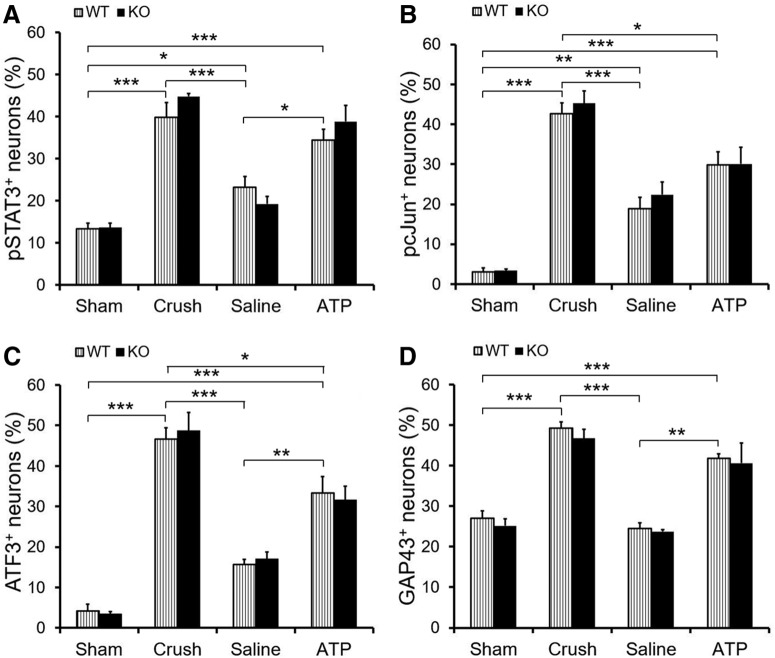
P2X7 receptor is not responsible for ATP-induced elevation of regenerative state of DRG neurons. P2X7R_KO mice were subjected to sham surgery, nerve crush, saline, and ATP injection. DRG were taken 3 d after treatments for immunohistochemistry. ***A***–***D***, Graphs showing the percentage of neurons with nuclear profiles containing immunoreactivity for pSTAT3 (***A***), pcJun (***B***), ATF3 (***C***), and GAP43 (***D***). *p < 0.05, **p < 0.01, ***p < 0.001. *n* = 6, two-way ANOVA with *post hoc* Tukey test.

P2X7R_KO mice were subjected to sham surgery, nerve crush, saline and ATP injection. L4–L5 DRG were taken 3 d after treatments. Immunohistochemistry results show that sciatic nerve crush significantly increased the expression of pSTAT3, pcJun, ATF3, and GAP43 in DRG neurons of both C57BL/6 wild-type and P2X7R_KO mice (Fig. 8). However, there is no significant difference between the wild-type and P2X7R_KO mice in all four molecular markers. Saline injection significantly increased the percentages of pSTAT3^+^ and pcJun^+^ neurons compared with the sham-operated group, whereas with injection of 1 mm ATP (a higher dose was used to activate P2X7R) significantly more neurons were pSTAT3^+^, ATF3^+^, and GAP43^+^ than those in saline-injected group. No difference was detected between the groups of wild-type and P2X7R_KO mice in both saline and ATP-injected groups. Also after T8 dorsal column transection, profile of regenerating axons of P2X7R_KO mice did not show significant difference with that of wild-type mice (data not shown). These results implicate that P2X7R may not be involved in sensory axon regeneration after conditioning lesion or intraneural ATP injection.

### Knocking out P2Y2 receptor reduces the number of pSTAT3^+^ DRG neurons.

**Figure 9.**
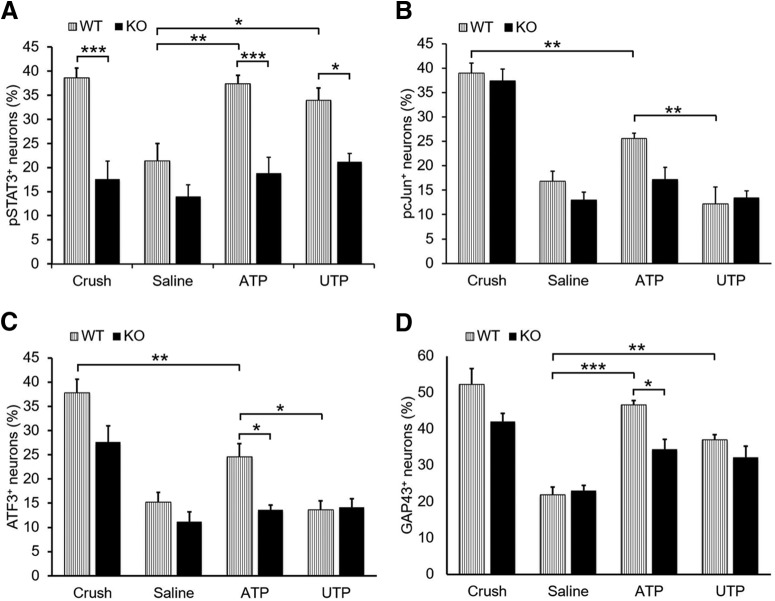
P2Y2 receptors are involved in ATP-induced elevation of regenerative state of DRG neurons. P2Y2R_KO mice were subjected to nerve crush, saline, UTP, and ATP injection. DRG were taken 3 d after treatments for immunohistochemistry. ***A***–***D***, Graphs showing the percentage of neurons with nuclear profiles containing immunoreactivity for pSTAT3 (***A***), pcJun (***B***), ATF3 (***C***), and GAP43 (***D***). *p < 0.05, **p < 0.01, ***p < 0.001. *n* = 4, two-way ANOVA with *post hoc* Tukey test.

To investigate the involvement of P2Y2R in conditioning lesion, apart from sciatic nerve crush and ATP injection, we also performed sciatic nerve injection of UTP, a relatively selective agonist for P2Y2R (Burnstock, 2012). Three days postinjection ATP (150 μm) or UTP (300 μm) significantly increased the percentage of pSTAT3^+^ DRG neurons of the wild-type mice compared with the saline-injected group (*p* = 0.006 and 0.05 respectively, *n* = 4; Fig. 9*A*). The percentages of pSTAT3^+^ neurons in P2Y2R_KO mice in crush, ATP, and UTP injection groups were significantly lower than the relevant wild-type groups (*p* = 0.0002, 0.001, and 0.045, respectively, *n* = 4), whereas there is no statistical significance between the P2Y2R_KO and the wild-type in the saline-injected group. These results indicate that P2Y2R plays a role in mediating STAT3 activation after conditioning lesion and ATP injection.

No significant difference in the percentages of pcJun^+^ neurons was detected between the P2Y2R_KO and the wild-type mice in all the four treatment groups although ATP injection increased the percentage of pcJun^+^ neurons more than UTP injection in the wild-type mice (Fig. 9*B*). Similarly, ATP injection increased the percentage of ATF3^+^ neurons than UTP injection in the wild-type mice (Fig. 9*C*). Significant difference was seen between the P2Y2R_KO and wild-type mice in ATP injection group, but not in UTP and crush groups. Both ATP and UTP injections also significantly increased the expression of GAP43 in DRG neurons in the wild-type mice compared with saline-injected mice (*p* = 2.1 × 10^−6^ and 0.004, respectively, *n* = 4; Fig. 9*D*). Again, ATP injection induced GAP43 expression in more neurons in the wild-type mice than in the P2Y2R_KO mice, whereas UTP injection did not.

### Intraneural ATP injection does not cause significant damage to the injected nerves

**Figure 10.**
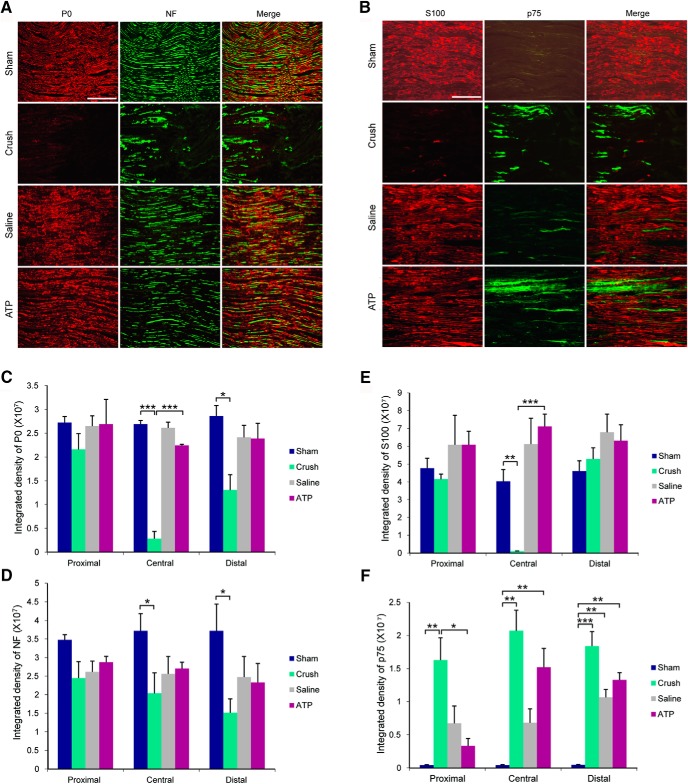
Histological changes of sciatic nerves after nerve crush or single intraneural injection of saline or ATP. Sciatic nerves were removed 3 d after treatments. ***A***, ***B***, Immunoreactivity of myelin protein P0 (***A***, red), axon marker NF 200 (***A***, green), Schwann cell marker S100 (***B***, red), and p75^NTR^ (***B***, green) in the central treatment region in sciatic nerves from the groups subjected to sham operation, nerve crush, injections of saline or ATP. Scale bars, 100 μm. ***C***–***F***, Quantification of the four markers in the proximal, central, and distal regions of the sites of treatments. *p < 0.05, **p < 0.01, ***p < 0.001. *n* = 3, one-way ANOVA with *post hoc* Tukey test.

As intraneural injection is an invasive procedure, we examined the histological changes at the injection sites of sciatic nerves. We immunostained for Schwann cell marker S100, peripheral myelin protein P0, neurofilament 200 (NF), and p75 neurotrophin receptor (p75^NTR^; a marker for dedifferentiated Schwann cells during Wallerian degeneration) in sections of nerves removed 3 d after sham operation, nerve crush, single saline, and single ATP injections. As expected, nerve crush caused significant reduction of P0, S100, and NF immunoreactivity at the crush center (Fig. 10). NF and P0 immunoreactivity in sciatic nerve distal to the crush site also reduced significantly, indicating the occurrence of Wallerian degeneration. S100 immunoreactivity in the distal sciatic nerve was not reduced, indicating that no significant Schwann cell death occurred. Nerve crush markedly increased p75^NTR^ immunoreactivity in the proximal, central, and distal regions of the nerve. Single injection of saline and ATP did not cause obvious changes in P0, S100, and NF immunoreactivity in the proximal, central, and distal injection sites, but did increase p75^NTR^ immunoreactivity. These observations indicate mild injury to axons in the injected nerves.

### Double injections do not cause long-term abnormality in sensory and motor functions

**Figure 11.**
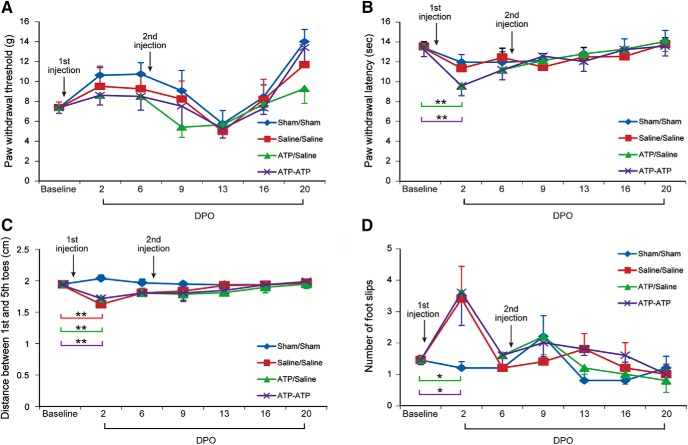
Effects of double intraneural injection of ATP on the sensory and motor function of sciatic nerves. ***A***, Mechanical sensitivity assessed by measuring paw withdrawal threshold using Von Frey hairs. ***B***, Noxious thermal sensitivity assessed using the Hargreaves' method. ***C***, Footprint analysis for the distance between the first and the fifth toe spread. ***D***, Grid walking test for the number of foot slips. DPO, Days post operation. *p < 0.05, **p < 0.01, compared with the baseline for the same group. *n* = 5, two-way ANOVA with *post hoc* Tukey test.

To exclude the possibility that intraneural ATP injection may affect sciatic nerve functions, we performed behavioral tests for the motor and sensory functions of the hindlimbs. Four groups of rats were subjected to tests for grid walking, hindpaw withdrawal upon mechanical or thermal stimulation, and footprint analysis after double sham operation or saline/saline, ATP/saline, and ATP/ATP double injections 1 week apart. Single and double injections of either saline or ATP did not cause significant change in mechanical thresholds of hindpaws during the 20 d testing period (Fig. 11*A*). Rats showed mild thermal hyperalgesia 2 d after the first ATP injection (Fig. 11*B*). The thermal withdrawal thresholds returned to baseline level 6 d later and did not significantly change after the second injection. Similarly, at day 2 after the first injection of either saline or ATP, the spread between the first and fifth toe was reduced (Fig. 11*C*), but returned to baseline level at day 6 and remained at the baseline level throughout the testing period. For grid walking test, rats from the injection groups made more footslips than normal on day 2, but recovered by day 6 (Fig. 11*D*). The results show that injection of ATP or saline caused mild injury to the sciatic nerve soon after injection; but that both sensory and motor functions recovered in a few days.

## Discussion

We have found that injection of ATP into a peripheral nerve can enhance regeneration of the corresponding central axons in the spinal cord and that two injections 1 week apart are far more effective than one, which supports our hypothesis that intraneural ATP injection can mimic the stimulatory effect of a conditioning lesion. ATP injection is also able to activate transcription factor STAT3 and increase the expression of GAP43 in DRG neurons in the same way as nerve injury does, further implicating that the release of endogenous ATP from injured cells may act as an injury signal, triggering the regenerative response of injured neurons. ATP has been implicated as an injury signal in brain and peripheral nerve injury. Local brain and spinal cord injury causes ATP release from damaged tissues (Wang et al., 2004; Franke et al., 2006) and the surrounding astrocytes, inducing rapid response of microglia toward injury (Davalos et al., 2005). Compression of peripheral nerve trunks resulted in immediate release of ATP (Grafe et al., 2006) and the released ATP is implicated to play a role in peripheral nerve degeneration and regeneration (Jung et al., 2014).

The links between the increased concentration of extracellular ATP in sciatic nerve after injection of ATP and the enhanced regenerative state of DRG neurons are still not fully understood. Because ATP injection activates STAT3 in DRG neurons, inducing the expression and release of neuropoietic cytokines may be a crucial step for the stimulatory effect on axonal growth. IL-6 is implicated in mediating the enhanced regenerative activity of DRG neurons after a peripheral nerve injury (Zhong et al., 1999; Cafferty et al., 2004). In this study we showed that a single ATP injection into sciatic nerve had a similar effect to nerve crush in increasing IL-6 expression in DRG and sciatic nerves (Bolin et al., 1995; Murphy et al., 1995). We also measured the concentrations of LIF and CNTF in sciatic nerves and DRG, however, the results were not informative due to the levels of LIF and CNTF were at the lower end of the detectable range.

Because ATP can be degraded rapidly in injected nerves, identification of the purinoceptor subtypes that are responsible for ATP-induced axonal growth may suggest the use of more potent and degradation-resistant agonists than ATP. Extracellular ATP can activate most of the seven P2X (ATP-gated cation channels) and eight P2Y (G-protein-coupled) purinoceptor subtypes. Furthermore, ATP is degraded to ADP, AMP, and adenosine and these purines can also activate purinoceptors such as P2Y1, P2Y12, and adenosine receptors (Burnstock, 2007). ATP is reported to induce transient increase of intracellular calcium in Schwann cells, a process mediated by P2Y receptors (Lyons et al., 1994) as well as P2X7 receptors (Luo et al., 2013). Because it is known that activation of P2X7R can lead to release of cytokines such as IL-1β (Ferrari et al., 2006), we postulated that it might also be involved in the release of IL-6 and LIF, leading to the activation of STAT3. However, no difference in the activation of the three transcription factors and upregulated expression of GAP43 was observed in sciatic nerve crush, ATP and saline-injected P2X7R_KO and the wild-type mice, which may exclude the involvement of P2X7R in the increased regenerative state of DRG neurons induced by conditioning lesion or ATP injection. UTP, an agonist for P2Y2 receptor subtype, was reported to release ATP from cultured Schwann cells (Liu et al., 2005). Injection of ATPγS, a nonselective agonist for several P2Y receptor subtypes, into sciatic nerve increased GAP43 expression in sciatic nerves of wild-type mice, but not in P2Y2R_KO mice (Arthur et al., 2005), indicating this receptor subtype may participate in ATP elevated regenerative state of DRG neurons. In this study, sciatic nerve crush, ATP, and UTP injection significantly activated STAT3 in DRG neurons in the wild-type mice, whereas the percentages of pSTAT3^+^ DRG neurons of P2Y2R_KO mice after the three treatments were significantly lower and not different from the saline-injected wild-type and P2Y2R_KO mice. These results indicate that P2Y2R is the P2 receptor subtype that is responsible for activation of STAT3. Using qPCR we have found that rat DRG, sciatic nerve, and cultured Schwann cells had high-level P2Y2R mRNA (unpublished data). P2Y2R proteins were expressed in DRG neurons, as detected by Western blot (Arthur et al., 2005). P2Y2R mRNA transcripts (Molliver et al., 2002) and proteins (Mo et al., 2013) were located in rat DRG neurons. We anticipated that P2Y2R was expressed by myelinating Schwann cells as well. However, we found that the anti-P2Y2R antibody we used (Alomone Labs) was not so specific since immunoreactivity was seen in DRG and sciatic nerves of P2Y2R_KO mice as well. Further studies on the cellular location of P2Y2R in sciatic nerves may shed light on the mechanisms of P2Y2R mediated axon growth. We have also identified the mRNAs of all other purinoceptor subtypes apart from P2Y11R in rat sciatic nerves, cultured Schwann cells, and DRG (unpublished data). It is likely that some other purinoceptor subtypes may also participate in the stimulatory action of ATP on axonal growth.

The data from this study also suggest that intraneural ATP injection could be developed into a clinically applicable treatment for overcoming one of the major obstacles to axonal regeneration in the adult mammalian CNS, namely poor intrinsic regenerative capacity. Although a conditioning lesion has been used frequently in animal models to promote sensory axonal regeneration as part of combinatory strategies (Zhang et al., 2007a,b; Kadoya et al., 2009; Blesch et al., 2012), it is not feasible to apply it clinically. In this study, we observed only mild histological changes in the injected nerves 3 d after injection and the adverse effects on the sensory and motor functions of the injected nerve subsided in a few days in the double injection experiments. We also examined the distribution of calcitonin gene-related peptide, substance P, and isolectin B4-positive fibers in the dorsal horn of lumbar spinal cord 20 d after double injections and did not observe significant sprouting of these nociceptive fibers (unpublished data). These findings support the observations from our behavioral tests that intraneural ATP injection does not induce hyperalgesia. On the other hand, a higher density of GAP43-positive fibers was observed in laminae III–V of the ipsilateral dorsal horn than the corresponding laminae in the contralateral dorsal horn in the ATP/saline and ATP/ATP injection groups (unpublished data). Laminae III–V are where the myelinated mechanoceptive axons project to, which corroborates the observation of enhanced regeneration of myelinated axons in the injured dorsal columns.

Our finding that a second ATP injection markedly boosted the stimulatory effect of a single injection mimicked what was reported by Neumann et al. (2005). Using a dorsal column transection model, they found that a second transection of the sciatic nerve a week after the first was able to promote ascending axons to grow across the lesion, whereas a single sciatic nerve transection did not. Repeated priming sustained the regenerative state of DRG neurons. It was shown previously that the percentage of pSTAT3^+^ DRG neurons after sciatic nerve transection dropped from 90% at 6 h postinjury to 36% 7 d postinjury, indicating that the conditioning effect may weaken in a week (Qiu et al., 2005). It is, therefore, not surprising that a second sciatic nerve transection or a second intraneural ATP injection 1 week later can boost the regeneration state of DRG neurons markedly. It is expected that repeated intraneural ATP injections may continue to sustain the regenerative state of DRG neurons; however, the procedure may not be practical. A different approach for repeated intraneural administration of ATP needs to be developed.

Because the axon growth promoting effect after double ATP injections was markedly more potent than a single ATP injection, one question raised is whether the needle injury of the first injection acted as a priming injury. Although we believe the effect of double ATP injections is mainly attributed to the biological actions of ATP, further study may be performed to exclude the possibility that a concomitant axon injury is required for the axon growth promoting effect of ATP.

In this study, spinal cord injury and ATP injection were performed concomitantly, which will not occur in clinical practice. Therefore, the efficacy of ATP injection in promoting sensory axonal regeneration needs to be tested in subacute and chronic injury animal models. A previous study showed that chronically injured DRG neurons acquire the intrinsic potential via conditioning lesion to regenerate their axons months after a CNS lesion (Ylera et al., 2009). We predict that intraneural ATP injection should also be able to elevate the regenerative state of DRG neurons in subacute and chronic dorsal column transection models. However, any successful therapeutic strategies will also have to deal with the poor cellular environment in the CNS for axonal regeneration and the challenge of directing axons to their original synaptic targets. Although we have demonstrated that double intraneural ATP injection can promote axon growth across the lesion epicenter, we anticipate that more profound axonal regeneration may be achieved if ATP injection is combined with other strategies such as blocking the myelin-associated inhibitors (Gonzenbach and Schwab, 2008; Wu et al., 2009), degrading chondroitin sulfate proteoglycans (Bradbury et al., 2002), overexpressing growth permissive molecules like polysialic acid (Zhang et al., 2007b), and providing cellular bridging using transplanted cells (Kadoya et al., 2009).

In conclusion, this study demonstrates that intraneural injection of ATP can elevate the regenerative state of DRG neurons and stimulate the growth of their central axons in injured spinal cord, whereas has no significant adverse effects on injected nerves, suggesting such an approach in stimulating sensory nerve regeneration is clinically translatable.

## References

[B1] Abbracchio MP, Burnstock G, Verkhratsky A, Zimmermann H (2009) Purinergic signalling in the nervous system: an overview. Trends Neurosci 32:19–29. 10.1016/j.tins.2008.10.001 19008000

[B2] Arthur DB, Akassoglou K, Insel PA (2005) P2Y2 receptor activates nerve growth factor/TrkA signaling to enhance neuronal differentiation. Proc Natl Acad Sci U S A 102:19138–19143. 10.1073/pnas.0505913102 16365320 PMC1323158

[B3] Blesch A, Lu P, Tsukada S, Alto LT, Roet K, Coppola G, Geschwind D, Tuszynski MH (2012) Conditioning lesions before or after spinal cord injury recruit broad genetic mechanisms that sustain axonal regeneration: superiority to camp-mediated effects. Exp Neurol 235:162–173. 10.1016/j.expneurol.2011.12.037 22227059 PMC3334479

[B4] Bolin LM, Verity AN, Silver JE, Shooter EM, Abrams JS (1995) Interleukin-6 production by Schwann cells and induction in sciatic nerve injury. J Neurochem 64:850–858. 10.1046/j.1471-4159.1995.64020850.x 7830079

[B5] Bradbury EJ, Moon LD, Popat RJ, King VR, Bennett GS, Patel PN, Fawcett JW, McMahon SB (2002) Chondroitinase ABC promotes functional recovery after spinal cord injury. Nature 416:636–640. 10.1038/416636a 11948352

[B6] Burnstock G (2007) Physiology and pathophysiology of purinergic neurotransmission. Physiol Rev 87:659–797. 10.1152/physrev.00043.2006 17429044

[B7] Burnstock G (2012) Purinergic signalling: its unpopular beginning, its acceptance and its exciting future. Bioessays 34:218–225. 10.1002/bies.201100130 22237698

[B8] Cafferty WB, Gardiner NJ, Das P, Qiu J, McMahon SB, Thompson SW (2004) Conditioning injury-induced spinal axon regeneration fails in interleukin-6 knock-out mice. J Neurosci 24:4432–4443. 10.1523/JNEUROSCI.2245-02.2004 15128857 PMC6729445

[B9] Chaplan SR, Bach FW, Pogrel JW, Chung JM, Yaksh TL (1994) Quantitative assessment of tactile allodynia in the rat paw. J Neurosci Methods 53:55–63. 10.1016/0165-0270(94)90144-9 7990513

[B10] Chessell IP, Hatcher JP, Bountra C, Michel AD, Hughes JP, Green P, Egerton J, Murfin M, Richardson J, Peck WL, Grahames CB, Casula MA, Yiangou Y, Birch R, Anand P, Buell GN (2005) Disruption of the P2X7 purinoceptor gene abolishes chronic inflammatory and neuropathic pain. Pain 114:386–396. 10.1016/j.pain.2005.01.002 15777864

[B11] Davalos D, Grutzendler J, Yang G, Kim JV, Zuo Y, Jung S, Littman DR, Dustin ML, Gan WB (2005) ATP mediates rapid microglial response to local brain injury *in vivo*. Nat Neurosci 8:752–758. 10.1038/nn1472 15895084

[B12] Dixon WJ (1980) Efficient analysis of experimental observations. Annu Rev Pharmacol Toxicol 20:441–462. 10.1146/annurev.pa.20.040180.002301 7387124

[B13] Ferrari D, Pizzirani C, Adinolfi E, Lemoli RM, Curti A, Idzko M, Panther E, Di Virgilio F (2006) The P2X7 receptor: a key player in IL-1 processing and release. J Immunol 176:3877–3883. 10.4049/jimmunol.176.7.3877 16547218

[B14] Fields RD, Burnstock G (2006) Purinergic signalling in neuron-glia interactions. Nat Rev Neurosci 7:423–436. 10.1038/nrn1928 16715052 PMC2062484

[B15] Franke H, Grummich B, Härtig W, Grosche J, Regenthal R, Edwards RH, Illes P, Krügel U (2006) Changes in purinergic signaling after cerebral injury: involvement of glutamatergic mechanisms? Int J Dev Neurosci 24:123–132. 10.1016/j.ijdevneu.2005.11.016 16387466

[B16] Gonzenbach RR, Schwab ME (2008) Disinhibition of neurite growth to repair the injured adult CNS: focusing on Nogo. Cell Mol Life Sci 65:161–176. 10.1007/s00018-007-7170-3 17975707 PMC11131900

[B17] Grafe P, Schaffer V, Rucker F (2006) Kinetics of ATP release following compression injury of a peripheral nerve trunk. Purinergic Signal 2:527–536. 10.1007/s11302-006-9018-y 18404490 PMC2096649

[B18] Hargreaves K, Dubner R, Brown F, Flores C, Joris J (1988) A new and sensitive method for measuring thermal nociception in cutaneous hyperalgesia. Pain 32:77–88. 10.1016/0304-3959(88)90026-7 3340425

[B19] He Z, Jin Y (2016) Intrinsic control of axon regeneration. Neuron 90:437–451. 10.1016/j.neuron.2016.04.022 27151637

[B20] Herdegen T, Fiallos-Estrada CE, Schmid W, Bravo R, Zimmermann M (1992) The transcription factors c-JUN, JUN D and CREB, but not FOS and KROX-24, are differentially regulated in axotomized neurons following transection of rat sciatic nerve. Brain Res Mol Brain Res 14:155–165. 10.1016/0169-328X(92)90170-G 1331648

[B21] Homolya L, Watt WC, Lazarowski ER, Koller BH, Boucher RC (1999) Nucleotide-regulated calcium signaling in lung fibroblasts and epithelial cells from normal and P2Y_2_ receptor (−/−) mice. J Biol Chem 274:26454–26460. 10.1074/jbc.274.37.26454 10473605

[B22] Hu-Tsai M, Winter J, Emson PC, Woolf CJ (1994) Neurite outgrowth and GAP-43 mRNA expression in cultured adult rat dorsal root ganglion neurons: effects of NGF or prior peripheral axotomy. J Neurosci Res 39:634–645. 10.1002/jnr.490390603 7534832

[B23] Jung J, Jo HW, Kwon H, Jeong NY (2014) ATP release through lysosomal exocytosis from peripheral nerves: the effect of lysosomal exocytosis on peripheral nerve degeneration and regeneration after nerve injury. Biomed Res Int 2014:936891. 10.1155/2014/936891 25101301 PMC4101216

[B24] Kadoya K, Tsukada S, Lu P, Coppola G, Geschwind D, Filbin MT, Blesch A, Tuszynski MH (2009) Combined intrinsic and extrinsic neuronal mechanisms facilitate bridging axonal regeneration one year after spinal cord injury. Neuron 64:165–172. 10.1016/j.neuron.2009.09.016 19874785 PMC2773653

[B25] Lee N, Neitzel KL, Devlin BK, MacLennan AJ (2004) STAT3 phosphorylation in injured axons before sensory and motor neuron nuclei: potential role for STAT3 as a retrograde signaling transcription factor. J Comp Neurol 474:535–545. 10.1002/cne.20140 15174071

[B26] Liu GJ, Werry EL, Bennett MR (2005) Secretion of ATP from Schwann cells in response to uridine triphosphate. Eur J Neurosci 21:151–160. 10.1111/j.1460-9568.2004.03831.x 15654852

[B27] Liu K, Tedeschi A, Park KK, He Z (2011) Neuronal intrinsic mechanisms of axon regeneration. Annu Rev Neurosci 34:131–152. 10.1146/annurev-neuro-061010-113723 21438684

[B28] Luo J, Lee S, Wu D, Yeh J, Ellamushi H, Wheeler AP, Warnes G, Zhang Y, Bo X (2013) P2X7 purinoceptors contribute to the death of Schwann cells transplanted into spinal cord. Cell Death Dis 4:e829. 10.1038/cddis.2013.343 24091672 PMC3824653

[B29] Lyons SA, Morell P, McCarthy KD (1994) Schwann cells exhibit P2Y purinergic receptors that regulate intracellular calcium and are up-regulated by cyclic AMP analogues. J Neurochem 63:552–560. 10.1046/j.1471-4159.1994.63020552.x 8035179

[B30] McQuarrie IG, Grafstein B (1973) Axon outgrowth enhanced by a previous nerve injury. Arch Neurol 29:53–55. 10.1001/archneur.1973.00490250071008 4711805

[B31] Mo G, Peleshok JC, Cao CQ, Ribeiro-da-Silva A, Séguéla P (2013) Control of P2X3 channel function by metabotropic P2Y2 UTP receptors in primary sensory neurons. Mol Pharmacol 83:640–647. 10.1124/mol.112.082099 23249537

[B32] Molliver DC, Cook SP, Carlsten JA, Wright DE, McCleskey EW (2002) ATP and UTP excite sensory neurons and induce CREB phosphorylation through the metabotropic receptor, P2Y2. Eur J Neurosci 16:1850–1860. 10.1046/j.1460-9568.2002.02253.x 12453048

[B33] Murphy PG, Grondin J, Altares M, Richardson PM (1995) Induction of interleukin-6 in axotomized sensory neurons. J Neurosci 15:5130–5138. 7623140 10.1523/JNEUROSCI.15-07-05130.1995 PMC6577897

[B34] Neumann S, Woolf CJ (1999) Regeneration of dorsal column fibers into and beyond the lesion site following adult spinal cord injury. Neuron 23:83–91. 10.1016/S0896-6273(00)80755-2 10402195

[B35] Neumann S, Skinner K, Basbaum AI (2005) Sustaining intrinsic growth capacity of adult neurons promotes spinal cord regeneration. Proc Natl Acad Sci U S A 102:16848–16852. 10.1073/pnas.0508538102 16275900 PMC1283855

[B36] Qiu J, Cai D, Dai H, McAtee M, Hoffman PN, Bregman BS, Filbin MT (2002) Spinal axon regeneration induced by elevation of cyclic AMP. Neuron 34:895–903. 10.1016/S0896-6273(02)00730-4 12086638

[B37] Qiu J, Cafferty WB, McMahon SB, Thompson SW (2005) Conditioning injury-induced spinal axon regeneration requires signal transducer and activator of transcription 3 activation. J Neurosci 25:1645–1653. 10.1523/JNEUROSCI.3269-04.2005 15716400 PMC6725934

[B38] Raivich G, Makwana M (2007) The making of successful axonal regeneration: genes, molecules and signal transduction pathways. Brain Res Rev 53:287–311. 10.1016/j.brainresrev.2006.09.005 17079020

[B39] Richardson PM, Issa VM (1984) Peripheral injury enhances central regeneration of primary sensory neurones. Nature 309:791–793. 10.1038/309791a0 6204205

[B40] Richardson PM, Verge VM (1987) Axonal regeneration in dorsal spinal roots is accelerated by peripheral axonal transection. Brain Res 411:406–408. 10.1016/0006-8993(87)91096-1 2440520

[B41] Richardson PM, Miao T, Wu D, Zhang Y, Yeh J, Bo X (2009) Responses of the nerve cell body to axotomy. Neurosurgery 65:A74–A79. 10.1227/01.NEU.0000352378.26755.C3 19927082

[B42] Rodrigues RJ, Tomé AR, Cunha RA (2015) ATP as a multi-target danger signal in the brain. Front Neurosci 9:148. 10.3389/fnins.2015.00148 25972780 PMC4412015

[B43] Schreyer DJ, Skene JH (1991) Fate of GAP-43 in ascending spinal axons of DRG neurons after peripheral nerve injury: delayed accumulation and correlation with regenerative potential. J Neurosci 11:3738–3751. 1836017 10.1523/JNEUROSCI.11-12-03738.1991 PMC6575281

[B44] Snider WD, Zhou FQ, Zhong J, Markus A (2002) Signaling the pathway to regeneration. Neuron 35:13–16. 10.1016/S0896-6273(02)00762-6 12123603

[B45] Tsujino H, Kondo E, Fukuoka T, Dai Y, Tokunaga A, Miki K, Yonenobu K, Ochi T, Noguchi K (2000) Activating transcription factor 3 (ATF3) induction by axotomy in sensory and motoneurons: a novel neuronal marker of nerve injury. Mol Cell Neurosci 15:170–182. 10.1006/mcne.1999.0814 10673325

[B46] Walker JL, Resig P, Guarnieri S, Sisken BF, Evans JM (1994) Improved footprint analysis using video recording to assess functional recovery following injury to the rat sciatic nerve. Restor Neurol Neurosci 6:189–193. 10.3233/RNN-1994-6303 21551749

[B47] Wang X, Arcuino G, Takano T, Lin J, Peng WG, Wan P, Li P, Xu Q, Liu QS, Goldman SA, Nedergaard M (2004) P2X7 receptor inhibition improves recovery after spinal cord injury. Nat Med 10:821–827. 10.1038/nm1082 15258577

[B48] Wu D, Yang P, Zhang X, Luo J, Haque ME, Yeh J, Richardson PM, Zhang Y, Bo X (2009) Targeting a dominant-negative rho kinase to neurons promotes axonal outgrowth and partial functional recovery after rat rubrospinal tract lesion. Mol Ther 17:2020–2030. 10.1038/mt.2009.168 19623163 PMC2814380

[B49] Ylera B, Ertürk A, Hellal F, Nadrigny F, Hurtado A, Tahirovic S, Oudega M, Kirchhoff F, Bradke F (2009) Chronically CNS-injured adult sensory neurons gain regenerative competence upon a lesion of their peripheral axon. Curr Biol 19:930–936. 10.1016/j.cub.2009.04.017 19409789

[B50] Zhang Y, Ghadiri-Sani M, Zhang X, Richardson PM, Yeh J, Bo X (2007a) Induced expression of polysialic acid in the spinal cord promotes regeneration of sensory axons. Mol Cell Neurosci 35:109–119. 10.1016/j.mcn.2007.02.011 17363265

[B51] Zhang Y, Zhang X, Wu D, Verhaagen J, Richardson PM, Yeh J, Bo X (2007b) Lentiviral-mediated expression of polysialic acid in spinal cord and conditioning lesion promote regeneration of sensory axons into spinal cord. Mol Ther 15:1796–1804. 10.1038/sj.mt.6300220 17551503

[B52] Zhong J, Dietzel ID, Wahle P, Kopf M, Heumann R (1999) Sensory impairments and delayed regeneration of sensory axons in interleukin-6-deficient mice. J Neurosci 19:4305–4313. 10341234 10.1523/JNEUROSCI.19-11-04305.1999 PMC6782624

